# Can surgical skills be taught using technological advances online? A comparative study of online and face-to-face surgical skills training

**DOI:** 10.1007/s00464-022-09170-5

**Published:** 2022-03-07

**Authors:** Matyas Fehervari, Bibek Das, Payam Soleimani-Nouri, Manal Ahmad, Michael G. Fadel, Mohammed Deputy, Catrin Morgan, Joshua R. Burke, John D. Mason, David Nott, Duncan Spalding

**Affiliations:** 1 Department of Surgery and Cancer, Faculty of Medicine, Imperial College London, London, UK; 2 Association of Surgeons in Training, London, UK; 3 London North West University Healthcare NHS Trust, London, UK; 4 Chelsea and Westminster Hospital, London, UK; 5 Surgical Epidemiology, Trials and Outcome Centre, St Mark’s Hospital and Academic Institute, London, UK

**Keywords:** Medical education, Surgical skills, Online teaching, Practical skills teaching, Validation

## Abstract

**Introduction:**

Online teaching has rapidly emerged as a viable alternative to traditional face-to-face education. How to teach surgical skills in the online environment, however, has not yet been fully established nor evaluated.

**Methods:**

An international 1-day online surgical skills course consisting of lectures, pre-recorded virtual workshops, live demonstrations and along with surgical skills teaching in breakout rooms was organised. Based on existing learning theories, new methods were developed to deliver skills teaching online. Simultaneously, traditional in-person surgical skills teaching was also conducted and used as a benchmark. Skills development was assessed by trained demonstrators and self-reported competency scores were compared between the online and face-to-face event.

**Results:**

553 delegates from 20 different countries attended the online course. Of these, 64 were trained in breakout rooms with a 1:5 demonstrator-to-delegate ratio whilst the remaining 489 delegates participated in didactic skills development sessions. In a separate face-to-face course, 20 delegates were trained with traditional methods. Demonstrators rated the competency of delegates for suturing, tendon repair and vascular anastomosis. There was no significant difference in the competency ratings of delegates receiving online teaching or face-to-face teaching (*p* = 0.253, *p* = 0.084, *p* = 1.00, respectively). The development of the same skills to “articulation” were not different between formats (*p* = 0.841, *p* = 0.792, *p* = 1.00, respectively). Post course self-rated competency scores improved for all technical skills (*p* < 0.001). Small group sessions, both online and face-to-face, received higher satisfaction ratings compared to large group sessions in terms of clarity of instructions, answers to questions and demonstrator feedback. Overall feedback on teaching quality, however, was equivalent across both groups.

**Discussion:**

Online teaching of surgical skills for early training years is an appropriate alternative to face-to-face teaching.

**Supplementary Information:**

The online version contains supplementary material available at 10.1007/s00464-022-09170-5.

## Introduction

Surgical training has been significantly impacted as elective procedures and hands-on training have been paused and later reduced during the last 2 years [1]. Face-to-face surgical courses have traditionally aimed to develop core skills and teach basic techniques used in surgical practice to enable delegates to safely perform these in clinical practice. These courses, however, have been on hold resulting in long waiting lists and a large number of trainees receiving limited practical skills teaching. Online teaching has emerged as an alternative as it offers the benefits of teaching simultaneously in different regions, reducing the cost, time and carbon footprint from travel and allows training in time of restrictions and social distancing [2]. There is currently limited information available on how basic surgical skills can be delivered effectively online [3, 4]. Online surgical training programmes have already begun [5] and validation of this novel method of surgical skills teaching is necessary.

Students can use different learning styles such as the VARK (visual, aural/auditory, read/write and kinaesthetic) model described by Fleming and Mills [6]. Learners with a preference for the first three elements of this model may benefit from didactic methods such as demonstration and description, hence online didactic delivery of skills teaching can be appropriate [5, 7, 8]. Kinaesthetic learners who prefer experience gained by performing those practical tasks can benefit from experimenting and practicing with synthetic models. In contrast, learning styles described by David Kolb identifies the cycle of accommodating, diverging, converging and assimilating and most surgical trainees vaccilate between these [9, 10]. Honey and Mumford also identified learners as activists, reflectors, theorists and pragmatist style learners [11]. Not surprisingly surgical trainees are most commonly activists or pragmatists who learn through practical tasks and experiments [12].

Online teaching of small groups can accommodate students with a preference towards practical learning using audio–visual demonstrations and remote supervision. This may be enhanced by the use of synthetic models. Surgical skills can be demonstrated to a camera followed by remote supervision as individuals practice on identical models [8].

Different teaching models based on these theories have not yet been evaluated and validated during online events. An international, 1-day virtual course was organised and evaluated whilst using face-to-face small group surgical skills teaching as a benchmark. Various learning styles were implemented by delivering different teaching methods, including supervised small group breakout rooms and large group didactic skills.

## Materials and methods

Junior surgical trainees and medical students were invited to a fully online Foundation Skills in Surgery Course to assess the development of basic surgical skills taught online. Learning outcomes of this course were compared to traditional face-to-face small group surgical skills teaching delivered simultaneously. The online course was advertised internationally on various social media platforms and electronic mailing lists. Face-to-face teaching was delivered in three UK Hospitals for local delegates. The primary endpoint of the study was the proportion of delegates reaching ‘articulation’ on Dave’s psychomotor domain, which we defined as students meeting ‘yes’ on all assessed domains for a particular skill. The secondary endpoint was self-reported skills development.

A specifically designed online platform able to simultaneously accommodate a virtual lecture hall and several breakout rooms was used (Hopin by Medall). The theory of organising the skill sessions was based on Dave’s taxonomy and progression of skills in the stages known as [13]; imitation, manipulation, precision, articulation and naturalisation. The aim was to develop the skills of the participants to the level of articulation or precision. This was further enhanced in the breakout rooms by the utilisation of Kolb and Fry’s theory of Experiential Learning [14], by progressing from concrete experience to reflective observation, abstract conceptualisation and finally active experimentation throughout learning.

Skills teaching was delivered online in breakout rooms and in the main lecture hall. Learning objectives were defined at the outset of each task. The skills were performed by two demonstrators to all delegates online in the ‘main lecture hall’. Pre-registered delegates were then admitted, again online, to breakout rooms whilst the others continued in the main lecture hall. In the breakout rooms demonstrators re-presented the skills and to facilitate imitation, the first domain of Dave’s taxonomy, asked students to instruct them throughout. In the next step, attendees started to practice by showing their model to the camera whilst demonstrators watched and corrected their techniques as necessary. In the main lecture hall, two demonstrators continued presentations of various surgical skills at a pace that students could practice alongside. Delegates could submit questions or requests to an online forum. At the end of the session breakout room participants were assessed by the demonstrators and provided with detailed feedback on their performance (Online Appendix 1). Breakout rooms were organised with a strict 1:5 demonstrator-to-delegate ratio. Demonstrators had to have completed medical school at least 5 years prior to the course and were required to be a Member of the Royal College of Surgeons. Demonstrators had to have training in teaching and providing feedback. They were required to attend a pre-course briefing where the exact teaching methods and delegate marking schemes were provided in a written format and discussed in detail.

Suitable teaching equipment consisting of commercially available models and surgical instruments were chosen in advance by the organising committee. A direct guide to purchase (with an electronic link) and assembly instructions were emailed to all delegates 4 weeks prior to the course. Any delegate who submitted a proof of purchase of the equipment required prior to the course was allowed entrance to breakout rooms. Participation in the course was free, but equipment had to be purchased and paid for by the delegates. The costs and exact description of equipment are described in Online Appendix 2. The study underwent an Educational Ethics Review Process which deemed formal ethical approval was not necessary.

Basic surgical skills were taught in the following order: scrubbing and theatre safety, surgical instruments, suturing and knot tying. Teaching of advanced techniques, such as vascular anastomosis and tendon repair occurred after the basic skills. Learning objectives were defined and demonstrated to all participants by two demonstrators prior to all workshops.

The mode of delivery of teaching individual skills was tailored to match previously described teaching methods and assessments to facilitate comparison of delegates performance [15–17]. The first three domains of the four-level Kirkpatrick evaluation method, including pre- and post-course questionnaires and training quality assessments, were used to evaluate the efficacy of the training sessions [18]. Translation to clinical practice was not assessed.

Questions that arose from students in the learning process were strongly encouraged and facilitated as per McAlpine et al. [19] as the process of knowledge assimilation and subsequent decision making was monitored and feedback provided throughout reflective practice was encouraged. Questions were answered directly by the demonstrators in breakout rooms. On the main stage, an online discussion forum was open to delegates and a moderator continuously monitored the forum for written questions and facilitated the discussion between the demonstrators and the online audience in the main lecture hall.

To determine whether online teaching could achieve the same outcomes as face-to-face teaching, we conducted a validation study comparing trainer competency ratings for delegates attending the online course, with a different skilled match cohort of students attending face-to-face teaching. All delegates, regardless of modes of teaching, were asked to complete a pre-course questionnaire (Online Appendix 3) and to provide post-course feedback via a link within an hour of the event finishing. Each skill (suturing, tendon repair and vascular anastomosis) was taught and assessed in several domains (Online Appendix 4). At the end of the online breakout room sessions and small group face-to-face teaching, demonstrators completed online assessment forms utilised in previous studies for each delegate on each taught skill [15–17]. Demonstrators and delegates completed the same questionnaires in all settings.

The level of skill development based on Dave’s Taxonomy was captured on these forms. After both the online and face-to-face skills sessions, trainers rated student competency on whether they achieved each domain with two possible outcomes: “Yes” or “No”.

### Statistical analysis

The sample size calculation was calculated using R and the package ‘SampleSize4ClinicalTrials’ using the following code: “install.packages("SampleSize4ClinicalTrials") library("SampleSize4ClinicalTrials") ssc_propcomp(design = 3L, ratio = 3, alpha = 0.05, power = 0.8, *p*1 = 0.90, *p*2 = 0.90, delta = 0.2)”. Assuming at least 90% of students meeting articulation criteria, our target sample size with a 3:1 allocation ratio was 57 students in the virtual group and 19 students in the face-to-face (control) group, with 80% power to demonstrate non-inferiority (alpha = 0.05 and a 20% non-inferiority margin).

Delegate skill ratings (0–10) before and after the course were compared using the Wilcoxon matched pairs signed-rank test. Delegates attending the breakout rooms and main lecture hall, respectively, rated the formats in several domains (0–10); which were compared using the Mann–Whitney *U* test. Categorical data were compared using the Chi-Square or Fisher’s exact test as appropriate. All statistical analyses were conducted using R software version 4.0.2.

## Results

**Fig. 1:**
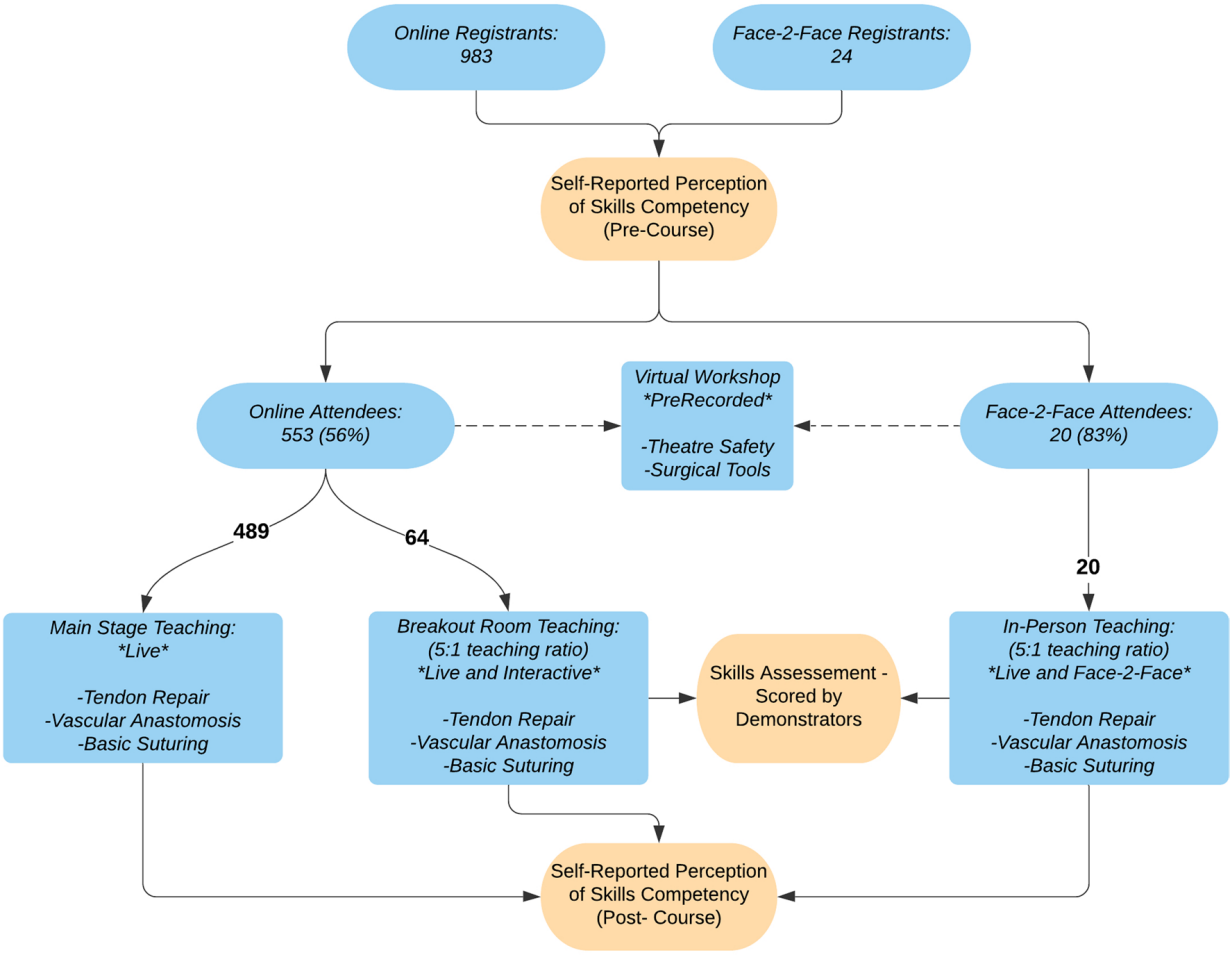
Structure of the skills session and flowchart to demonstrate the online attendance and in-person attendance

There were 983 delegates registered onto the online course with an overall attendance of 553 (56%). A total of 24 delegates registered for face-to-face teaching and 20 of them attended (86%). Delegates attended from 20 different countries on the course. The number of delegates from each country is listed and displayed in Online Appendix 5. A flowchart of the course structure is presented in Fig. 1.

Delegates spent on average 359 min on the online course and submitted 2579 comments (mean of 4.6 comments per delegate). Most of the attendees were medical students (*n* = 307, 79%), with many in their final year of study (*n* = 100, 27%), followed by 62 (17%) qualified doctors in postgraduate years 1–4. Sixty-four (17%) delegates attended an online breakout room: 41 (64%) medical students and 23 (36%) qualified doctors. All delegates in breakout rooms used the models suggested by the course organisers. The skilled matched cohort on the face-to-face event consisted of 14 final-year medical students (70%) and 6 qualified doctors (30%).

**Fig. 2:**
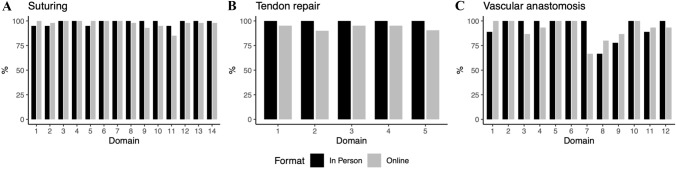
Delegate competency ratings by trainers after face-to-face or online teaching. Percentage reflects the proportion of students meeting each domain after each teaching format. The X-axis is the domain for each skill further detailed in the supplementary material. The Y-axis is the percentage of competency achievement in that domain for each skill. Full details of assessment domains are provided in the supplementary material

Evaluation of small group online and face-to-face teaching suggested no difference between demonstrator or self-reported skills development. After each session competency ratings submitted by demonstrators were equal in all individual domains between delegates undergoing online or face-to-face teaching and assessment (Fig. 2).

A summative score was developed by merging individual domains into a single score for each taught skill. Comparing these summative scores for suturing, tendon repair and vascular anastomosis showed no difference between online and face-to-face teaching (*p* = 0.253, *p* = 0.084 and *p* = 1.00, respectively). The development of suturing skills, tendon repair and vascular anastomosis to the level of articulation were no different between delegates participating in breakout rooms compared to face-to-face sessions (*p* = 0.841, *p* = 0.792 and *p* = 1.00, respectively).

**Fig. 3:**
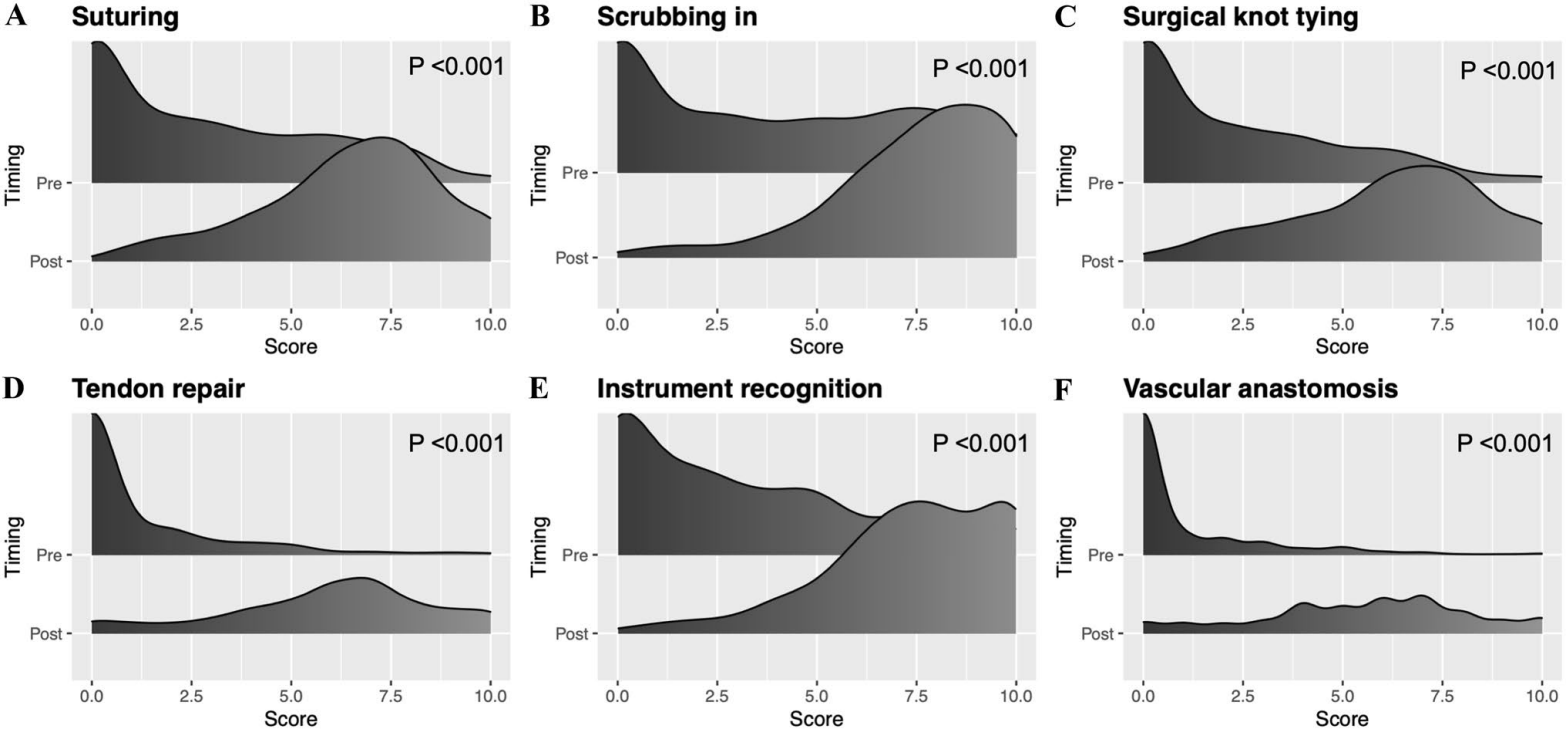
Self reported confidence ratings by the participants before and after the virtual conference in each of the various skills including suturing, scrubbing-in, surgical knot tying, tendon repair, instrument recognition and vascular anastomosis. The x-axis is the score from 0 to 10 reported by participants. The Y-axis demonstrates the score for each skill before and after the teaching

Overall self-rated competency scores improved for all technical skills (*p* < 0.001) at the completion of the course (Fig. 3). Delegates did not report a significant change in preference for either online or face-to-face teaching (*χ*^2^ = 0.24, *p* = 0.63) and reported feeling more confident with applying for higher surgical training (*p* < 0.001) and were more likely to pursue a surgical career (*p* = 0.006) after the course.

Overall, the vast majority of participants strongly agreed that the level of the course and the learning outcomes were clearly defined (77%, 80%), delivery methods were appropriate (76%), it was conducted in a suitable environment (67%), had an appropriate structure (84%), and had a sufficient number of demonstrators (84%) who presented appropriate knowledge and skills for the learning outcomes (89%). Most were highly likely (> 7/10) to recommend the course to a friend (90%) and were satisfied with the platform (92%).

**Fig. 4:**
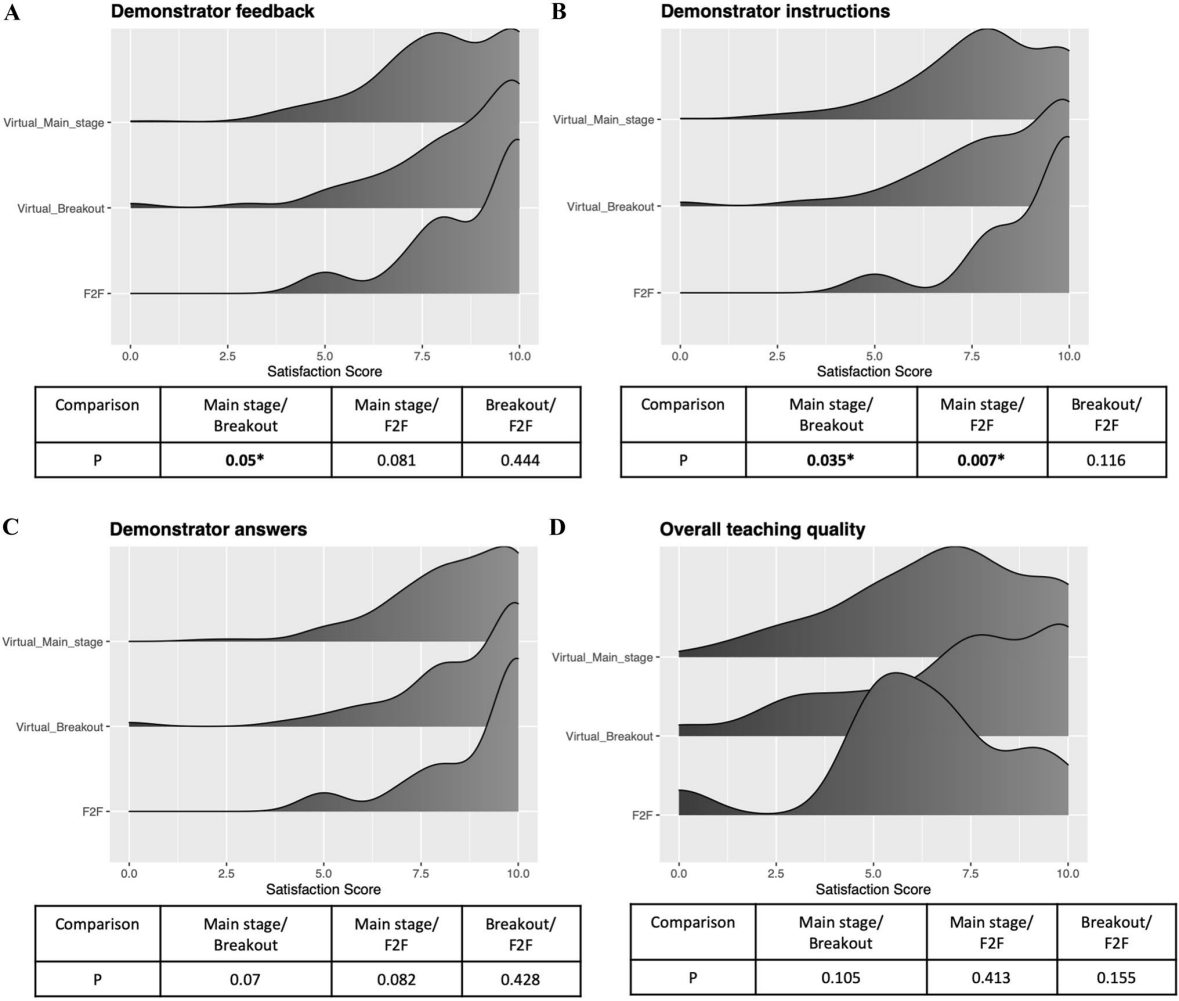
Satisfaction ratings from delegates on the x-axis (range 0-10) attending breakout rooms, the main stage on the virtual platform and face-to-face sessions on the Y-axis (* implies difference was significant) in the overall Demonstrator Feedback, Demonstrator Instructions, Demonstrator answers and overall teaching quality

Comparing the three different deliveries of teaching suggested higher satisfaction ratings from small group teaching regardless of whether it was online or face-to-face compared to didactic main stage lectures in terms of demonstrator feedback (median score 9/10 vs. 10/10 vs. 8/10), clarity of instructions (median score 9/10 vs. 10/10 vs. 8/10) and answers from demonstrators to questions (median score 9/10 vs. 10/10 vs. 9/10). When comparing online breakout room sessions to face-to-face teaching with the same (1:5) demonstrator to delegate ratio, no difference was found in these domains. Overall teaching quality was similar across the three types of teaching, online teaching quality matched that of a face-to-face session (*p* = 0.11) (Fig. 4).

## Discussion

This was the first online, large-scale, surgical skills course comparing various online teaching models based on previously described learning styles, with subsequent validation against the gold standard of face-to-face teaching [6, 14]. In many undergraduate and postgraduate teaching centres, online education has now become the standard, however, teaching of practical surgical skills has not yet been developed, widely implemented or evaluated [20, 21].

An exceptionally high number of delegates registered and attended the virtual event which was achieved through advertising and high-quality lectures. Delegates on the course consisted of a broad range of trainees, from beginners to doctors with up to 3 years of surgical training. The average length of time spent on the course and the written comments per delegate is evidence of a high level of engagement of all parts of the course.

Specifically trained demonstrators assessed using established marking schemes and self-reported skills development suggested that skills matched cohort of delegates achieved equivalent levels of surgical skills following both online and face-to-face small group teaching. The majority of delegates felt that online breakout room teaching matched the quality of face-to-face teaching. Small group surgical skills teaching has been previously shown to have the ability to increase confidence in key surgical skills and help to develop them to the level of ergonomic efficiency [22]. Our findings suggest similar outcomes describing equal skills development to the level of articulation in both online and face-to-face cohorts. These findings validate this novel way of surgical skills training against the gold standard of face-to-face teaching. Also, we suggest that the kinaesthetic or practical element of acquiring a surgical skill relates to the individual’s own tactile experience with the instrument and the model, rather than the trainer’s guidance.

Self-reported development was equal between the online main lecture hall teaching and breakout room sessions, regardless of the difficulty of taught skills and the level of previous training. There was marginally higher satisfaction with the quality of teaching reported by online breakout room and face-to-face attendees compared to main lecture hall delegates.

Delegate feedback suggests that this event met the required standards of a high-quality surgical teaching course. Despite the course focussing on practical surgical skills delegates did not feel that face-to-face teaching would have been more appropriate.

Comparing the three modes of teaching delivery suggested that small group teaching, regardless of whether it was online or face-to-face, was a more appropriate method of teaching surgical skills. There was an improvement, however, in self-reported skills development by delegates attending the main stage. Of note, a higher score was recorded for clarity of instructions in the breakout rooms when compared to the main lecture hall.

There are some limitations to this study. The retention of these skills after online teaching is important and has not been assessed. Lecture-based online teaching will also require validation beyond self-reported development.

Small group online teaching for early training years is an appropriate method to teach surgical skills, both at a basic and advanced level. It is important to design or suggest equipment that is easy to use and readily available. Trainees can learn from their individual practice which can be effectively supervised online. Retention of skills by using more detailed numeric scoring systems as an assessment method, however, will need to be assessed in future studies. Lecture-based online skills teaching with written questions provides some benefit in learning surgical skills and has the additional advantage of teaching a large number of delegates simultaneously. This may be an appropriate first step in surgical skills teaching in the first years of medical school. In summary, online teaching is comparable to traditional face-to-face methods and may be used for surgical skills development of junior trainees and medical students in the future.

## Supplementary Information

Below is the link to the electronic supplementary material.

Supplementary file1 (PDF 1570 kb)

Supplementary file2 (PDF 228 kb)

Supplementary file3 (PDF 52 kb)

Supplementary file4 (PDF 427 kb)

Supplementary file5 (PDF 266 kb)
