# Supplementary material for: Can surgical skills be taught using technological advances online? A comparative study of online and face-to-face surgical skills training
Source: Surg Endosc. 2022 Mar 7;36(6):4631–7. doi: 10.1007/s00464-022-09170-5 (PMC9085701; doi:10.1007/s00464-022-09170-5)
Supplement: Supplementary file 1 — Supplementary file1 (PDF 1570 kb) [file 464_2022_9170_MOESM1_ESM.pdf]

Imperial College Surgical Society and
The Association of Surgeons in Training

are proud to present

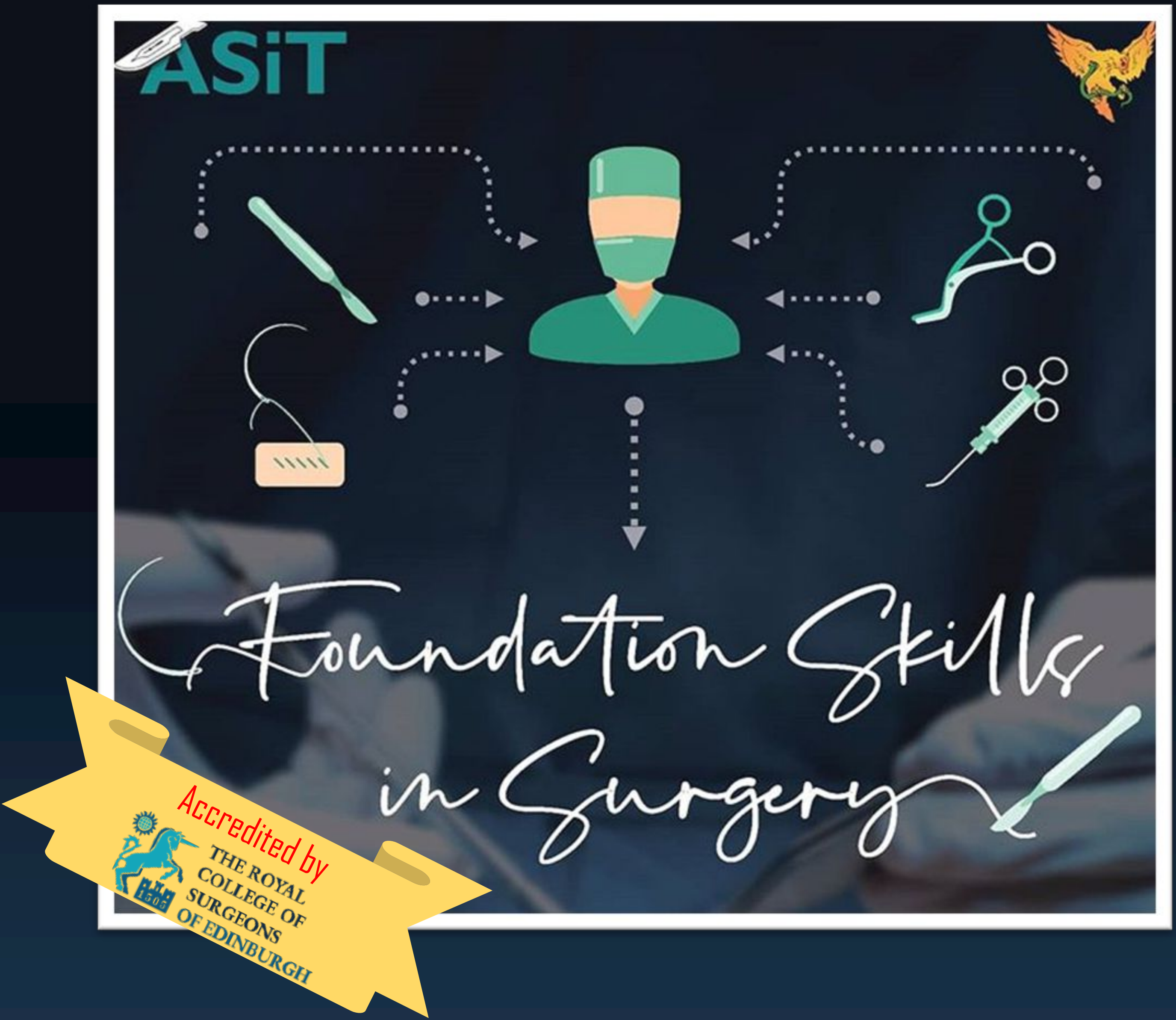

Delegates Booklet

Featuring the highest quality:

- Lectures
- Basic Surgical Skills Workshops
- Specialist Surgical Workshops

Saturday 13th February 2021
Virtual Conference
Imperial College London

TABLE OF CONTENTS

2 **WELCOME**
Opening Message

3 **EVENT TIMETABLE**
Foundation Skills in Surgery Synopsis

5 **SPEAKERS**
Imperial College London, Surgical Society

6 **WORKSHOPS**
Opening Message

7 **SPONSORS**
Opening Message

9 **THE SOCIETY AND PATRONS**
Imperial College London, Surgical Society

11 **CLOSING REMARKS**
Thank You

Welcome!

Imperial College Surgical Society is proud to announce that it will be hosting its annual Foundation Skills in Surgery (FSS) course this year on the **13th February 2021**, virtually on Hopin.

Run in collaboration with the Association of Surgeons in Training (ASiT), the **international** course brings together over a **dozen** surgeons to an event where they share their expertise and skills with **hundreds** of **delegates** ranging from medical students to qualified doctors. The course aims to develop delegates into confident and skilled surgeons of the future. Lectures, delivered by pioneering surgeons and accomplished surgical trainees, will be delivered on surgical careers, surgical CVs, and 'hostile-surgery'. FSS will prepare its participants to excel in the surgical field both inside and outside the operating room.

At FSS 2021, we will welcome a range of distinguished speakers who will share their expertise in the field of surgery in order to assist and motivate you in your own pursuit of a surgical career. Speakers will include the likes of **Prof David Nott OBE**, the award winning author of best selling memoir 'War Doctor' and **Prof George Hanna**, head of the division of Surgery and Cancer at Imperial College London.

As the day progresses, we will demonstrate basic surgical skills as outlined by the Royal College of Surgeon's syllabus on BSS training. These workshops will include tutorials on **theatre etiquette**, **basic suturing techniques**, **knot tying** and even more advanced procedures such as **tendon repair** and **anastomosis**.

We sincerely hope that FSS 2021 will be an inspirational experience for you and that it will be a boosting moment in your long and prosperous career as a surgeon.

With warm regards,

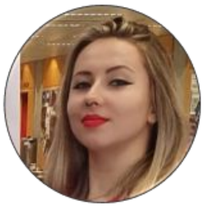

Julia Komor
ICSM Surgical Society Co-Chair 2020-2021

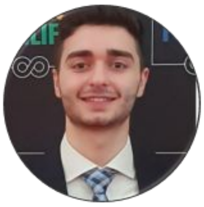

Payam Soleimani-Nouri
FSS Course Lead

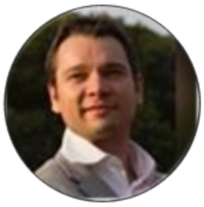

Matyas Fehervari
ASiT NW London Rep
Course Lead

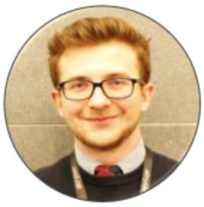

Michal Kawka
ICSM Surgical Society Co-Chair 2020-2021

Foundation Skills in Surgery Timetable 2021

Imperial College London Surgical Society | Association of Surgeons in Training

Time	Title	Description	Host
8:30		**Registration**	
9:00 – 9:10	**Introduction**	Opening Speech	*Payam Nouri*<br>*Mr Matyas Fehervari*
9:10 – 9:50	**Lecture 1**	How to ACE your Surgical Trainee Portfolio	*Mr Ankur Khajuria*
10:00 – 10:40	**Lecture 2**	Neuroergonomics and Neuroenhancement	*Mr Daniel Leff*
10:40 – 11:10	**Virtual Workshop**	Surgical Tools: Diathermy	*Mr Matthew Hide*
11:10 – 11:30		**Networking Break**	
11:30 – 12:30	**Virtual Workshop - BSS**	A. Surgical suturing<br>B. Surgical Hand-tying	*Various*
12:30 – 13:00		**Lunch Break**	
13:10 – 14:00	**Lecture 3 - Keynote**	Surgery in the Most Hostile of Environments	*Prof David Nott*
14:00 – 14:15	**Virtual Workshop**	Theatre Safety	*Ms Manal Ahmad*
14:15 – 15:00<br>15:00 – 15:45	**Practical Workshop – Specialist**	A. Repair of Torn Tendon<br>B. Vascular Anastomosis	*Various*
15:45 – 16:00		**Networking Break**	
16:00 – 16:40	**Lecture 4 - Keynote**	Surgery: An Art or a Science	*Prof George Hanna*
16:40 – 16:45	**Prize Giving**	Awards given to best surgical skills	*Payam Nouri*
16:45 – 16:50	**Closing Statement**	Evaluation of Course	*Payam Nouri*<br>*Mr Matyas Fehervari*

Brought to you by

Proudly Sponsored by

Accredited by

Event Synopsis

Lectures

- Delivered by **industry leading** academics and surgeons
- Give insight and tips on the **application process** of Core Surgical Training. CV-building advise will be provided to delegates.
- Provide a foundation to **Basic Surgical Instruments** and critical information to know about them before entering theatre

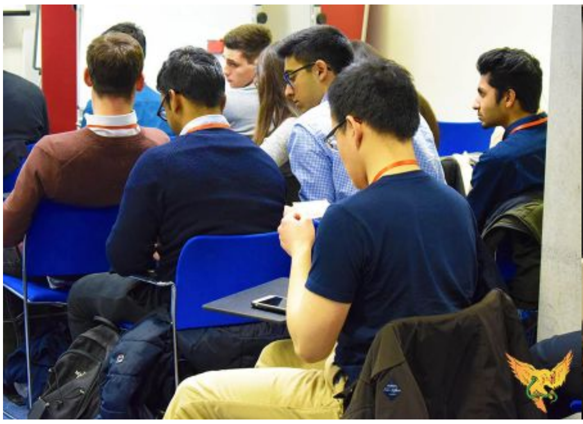

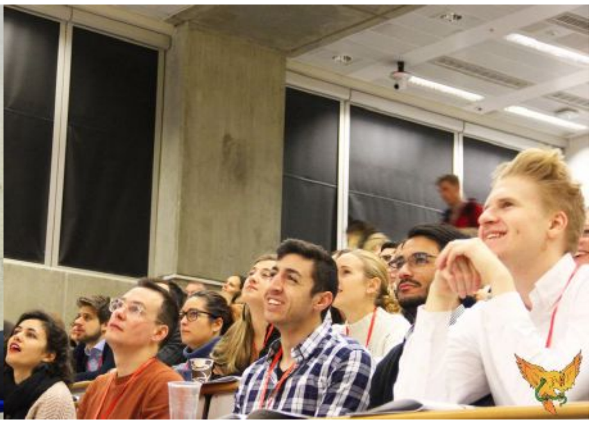

Practical Workshops

- Introducing delegates to **Basic Surgical Skills** as outlined by the **RCS** syllabus. Including: suturing, knot-tying, hand-washing, gowning and gloving
- Advanced surgical techniques will also be provided including: **tendon repair** and vascular **anastomosis**

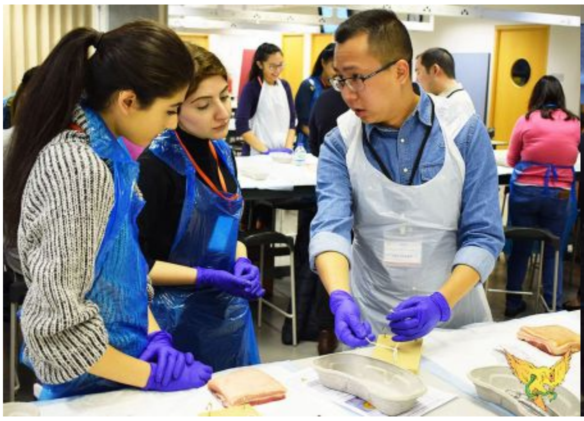

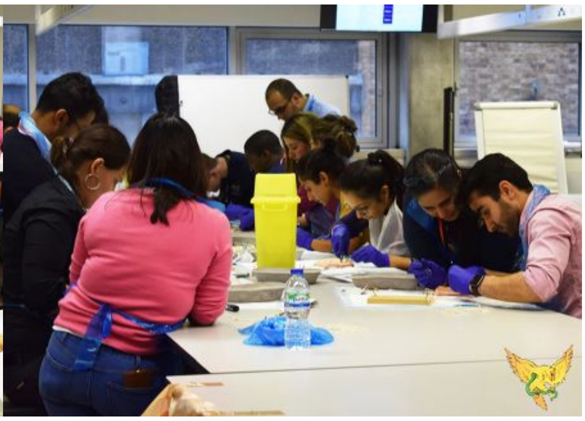

Brought to you by

Proudly Sponsored by

Accredited by

Speakers

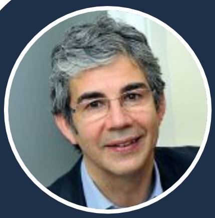

Keynote

Prof David Nott OBE

Consultant General Surgeon

David Nott is a Consultant Surgeon at St Mary's Hospital where he specialises in vascular and trauma surgery. For the past twenty five years Prof Nott has taken unpaid leave each year to work for the aid agencies Médecins Sans Frontières, the Red Cross and Syria Relief. Prof Nott is also the author of the bestselling memoir, War Doctor.

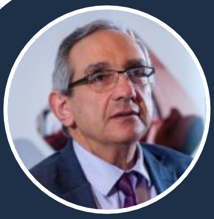

Keynote

Prof George Hanna

Consultant General Surgeon

Prof Hanna is a general surgeon with special interests in keyhole, laparoscopic and oesophago-gastric surgery. He is a Professor of Surgical Sciences and Head of the Surgery at Imperial College London. Prof Hanna's research includes the development of a non-invasive breath test to diagnose oesophageal and gastric cancer, and the development of a radiofrequency-based system for bowel anastomosis.

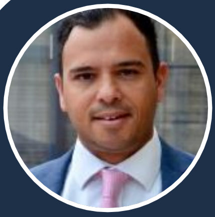

Mr Daniel Leff

Consultant Oncoplastic Surgeon

Mr Leff is a Reader in breast surgery working in the departments of biosurgery and surgical technology, the Hamlyn Centre for Robotic Surgery and the Cancer Research UK Centre at Imperial College London. He is an honorary consultant in oncoplastic breast surgery at Imperial College Trust.

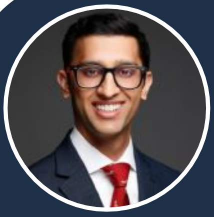

Mr Ankur Khajuria

Plastic & Reconstructive Surgeon

Mr Khajuria is the founder and chairman of High Yield UK. He ranked 1st in the country in the UK Plastic Surgery National Selection. He is also a Kellogg Research Scholar at Oxford University, Honorary Research Fellow at the Institute of Global Health Innovation at Imperial College London

Brought to you by

ASiT

Proudly Sponsored by

MDU WESLEYAN we are all about you Royal College of Surgeons BMA Swann-Morton Rocialle STORZ KARL STORZ — ENDOSKOPE

Accredited by

THE ROYAL COLLEGE OF SURGEONS OF EDINBURGH

Workshops

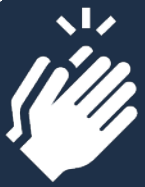

Hand-washing, Gowning and Gloving to a Surgical Standard

Mapped Learning Outcome:
On completion of this course, delegates should be able to demonstrate a proficient and safe entrance into the theatre by washing hands, gloving and gowning to a surgical standard in a simulated environment.

Workshop Contents:
- Surgical Hand Washing
- Surgical Gloving
- Surgical Gowning

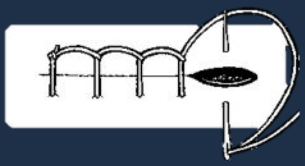

Surgical Suturing and Knot tying

Mapped Learning Outcome:
On completion of this course, delegates should be able to perform at least one type of basic suture (interrupted, continuous, mattress, cruciate or subcuticular) with confidence in a simulated environment, and perform a surgical hand knot (Aberdeen style) in a simulated environment

Workshop Contents:
- Surgical Suturing
- Surgical Knot Tying

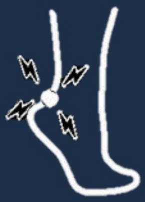

Repair of a Torn Tendon

Mapped Learning Outcome:
On completion of this course, delegates should be able perform a specialist procedure such as tendon repair

Workshop Contents:
- Repair of Torn Tendon

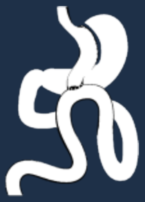

Surgical Anastomosis

Mapped Learning Outcome:
On completion of this course, delegates should be able perform a specialist procedure such as a surgical anastomosis

Workshop Contents:
- Surgical Anastomosis

Brought to you by

Proudly Sponsored by

Accredited by

Our Sponsors

Swann-Morton
- Swann-Morton are a world leader in the manufacture of surgical blades, scalpels and handles.
- The preferred choice of many of the World's surgeons
- Exporting to over 100 countries around the globe

The British Medical Association (BMA)
- Trade union and professional body for doctors in the UK.
- Represents, supports and negotiates on behalf of all UK doctors and medical students.

Medical Defence Union (MDU)
- The leading medical defence organisation in the UK.
- Provide professional medical indemnity to doctors and other healthcare professionals

The Royal College of Surgeons England (RCSEng)
- Organisation which exists to advance patient care in surgical setting.
- Support over 25,000 members by improving their skills and knowledge, facilitating research and developing policy.

Our Sponsors

Association of Surgeons in Training (ASiT)

- Aim of promoting excellence in surgical training.
- Represents over 2,700 surgical trainees at both regional and national levels in the United Kingdom and Ireland.

Rocialle

- For over thirty years, Rocialle has supplied healthcare providers with sterile and non-sterile consumable items, vital to patient care.

Karl-Storz

- Since 1945, the KARL STORZ family company has grown into a global manufacturer and distributor of endoscopes, medical instruments, and devices.

Wesleyan

- Wesleyan provides personal income protection to a wide range of professionals including medical professionals should you become unable to continue your medical studies or work due to an illness or injury.

Who are we?

The University – Imperial College London

- Ranked **3rd in Europe** and 4th in the world for 'clinical, preclinical and health sciences' (Times Higher Education World University Rankings 2018)
- Ranked **8th in the world** (QS World University Ranking 2019)
- Imperial College School of Medicine (ICSM) ranked 5th in the UK for Medicine (Complete University Guide 2019)

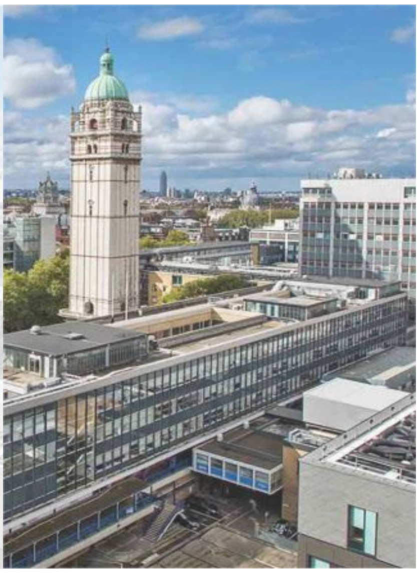

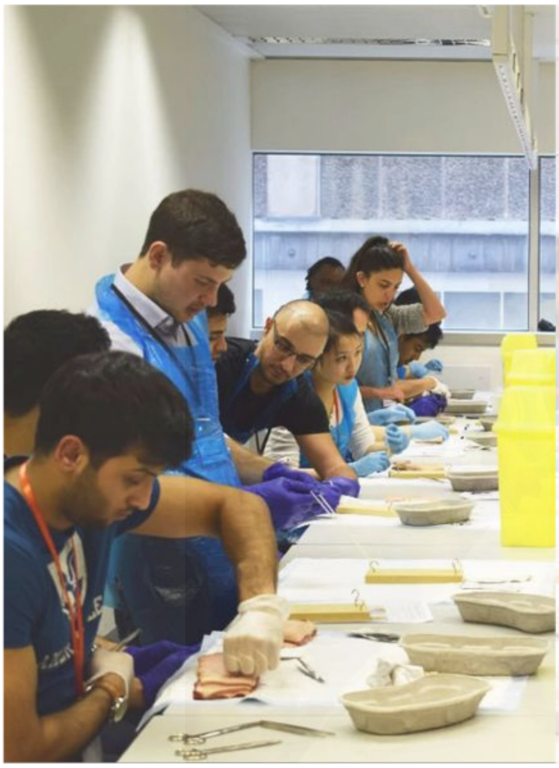

The Society – ICSM Surgical Society

- Formed in **2005** to elevate participation and recognition of medical students in extracurricular surgical training
- Partnered with the **Royal College of Surgeons** of England
- **Highly commended** by the Royal College of Surgeons of Edinburgh in **2012**. Winner of the Imperial College Union President's Award in 2008
- Annually hosts a plethora of popular, educational events

Patrons

of Imperial College Surgical Society

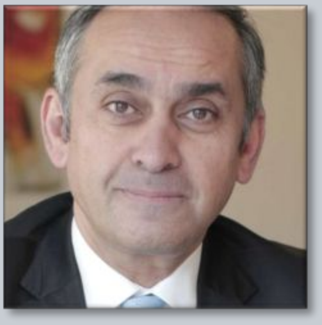

Professor Lord Ara Darzi of Denham
KBE, FMedSci, HonFREng, FRCS, FRCSI, FRCSed, FRCPSG, FACS, FCGI, FRCPE

- UK Health Minister 2007-2009
- Consultant Surgeon, St. Mary's Hospital
- Head of Division of Surgery, Oncology, Reproductive Biology and Anaesthetics, Imperial College London
- Paul Hamlyn Chair of Surgery, Imperial College London

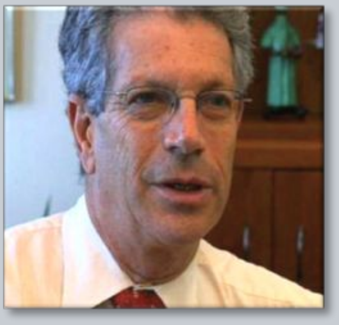

Professor Kenneth D. Boffard
BSc, MBChB, FRC (Ed), FRCS (Eng), FRCPS (Glas), FCS (SA), FACS

- Emeritus Professor of Trauma Surgery, Johannesburg Hospital
- Director of Trauma, Milpark Hospital Trauma Centre
- President of International Society of Surgery
- Governor (SA) of the American College of Surgeons
- President of South Africa Trauma Society

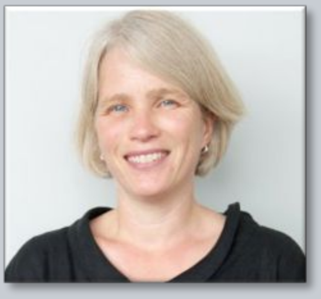

Professor Alison McGregor
PhD, MSc, MCSP

- Professor of Musculoskeletal Biodynamics, Imperial College London
- Associate director of Imperial College London
- London's Centre for Blast Injury Studies
- Past President of the Society for Back Pain Research
- Executive of the International Society for the Study of the Lumbar Spine

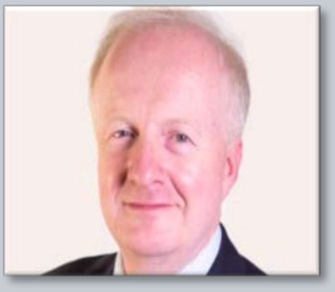

Professor Alun H Davies
MA, DM, DSC, FRCS, FHEA, FEBVS, FACPH

- Professor of Vascular Surgery, Imperial College London
- Director of the European College of Phlebology
- Past president of the European Venous Forum
- Editor-in-chief of Phlebology
- Previous Chairman of Varicose Vein Guideline Group for the National Institute of Clinical Excellence (NICE)

Closing Remarks

We would like to thank you for your attendance at FSS 2021, a surgical skills conference held by Imperial College Surgical Society in collaboration with the Association of Surgeons in Training.

If you have further questions in relation to this course, please contact the representatives below:

Payam Nouri
ICSM Surgical Society
Course Lead, FSS

E: ps1817@ic.ac.uk

Matyas Fehervari
ASiT Representative,
North West London

E: matyas.fehervari15@imperial.ac.uk

Julia Komor
ICSM Surgical Society,
CoPresident

E: julia.komor15@imperial.ac.uk

For more information about ICSM Surgical Society, please visit our **social media** pages or website https://www.imperialsurgicalsociety.co.uk

/ICSMSurgicalSociety @ICSMSurgicalSoc /ICSMSurgicalSoc

We look forward to hearing from you soon.

With best wishes,
ICSM Surgical Society

ICSM SURGICAL SOCIETY

Foundation Skills in Surgery
2021

Imperial College Surgical Society
Association of Surgeons in Training
