# Supplementary material for: Can surgical skills be taught using technological advances online? A comparative study of online and face-to-face surgical skills training
Source: Surg Endosc. 2022 Mar 7;36(6):4631–7. doi: 10.1007/s00464-022-09170-5 (PMC9085701; doi:10.1007/s00464-022-09170-5)
Supplement: Supplementary file 2 — Supplementary file2 (PDF 228 kb) [file 464_2022_9170_MOESM2_ESM.pdf]

## Station 1
Suturing and Knot Tying

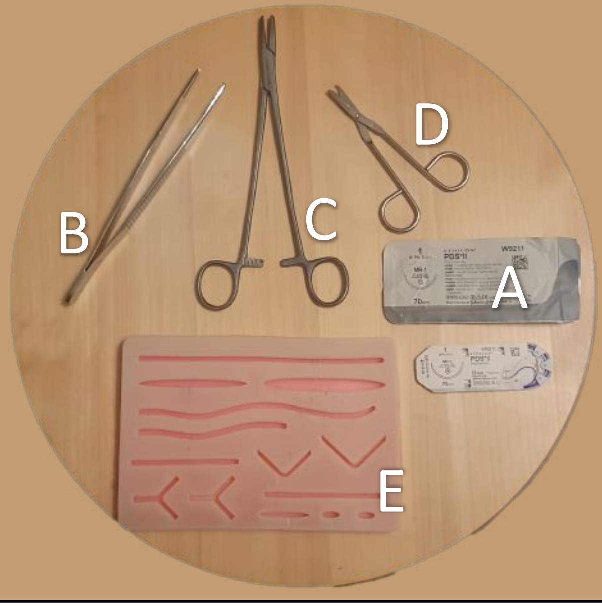


**Equipment:**

A. Sutures (preferably Prolene 3.0)
B. Toothed Forceps
C. Needle Holder
D. Suture Scissors

E. Skin Pad

## Station 2
Tendon Repair

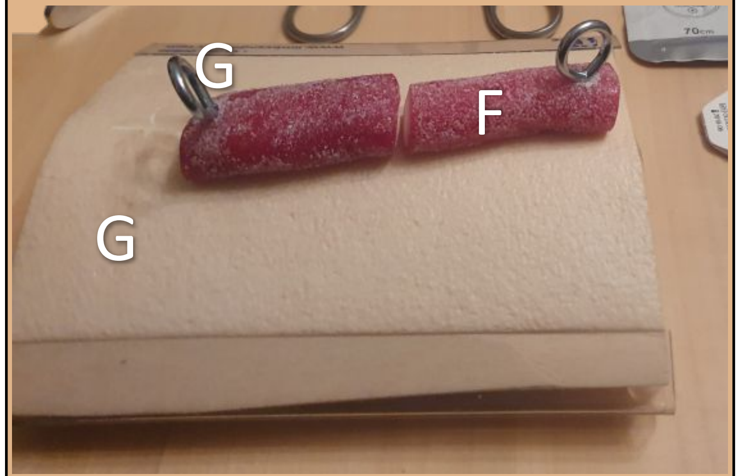


**Equipment:**

A. Sutures (preferably Prolene 3.0)
B. Toothed Forceps
C. Needle Holder
D. Suture Scissors

F. Haribo Stixx
G. Pad + Pins (to pin down Haribo)

## Station 3
Vascular Anastomosis

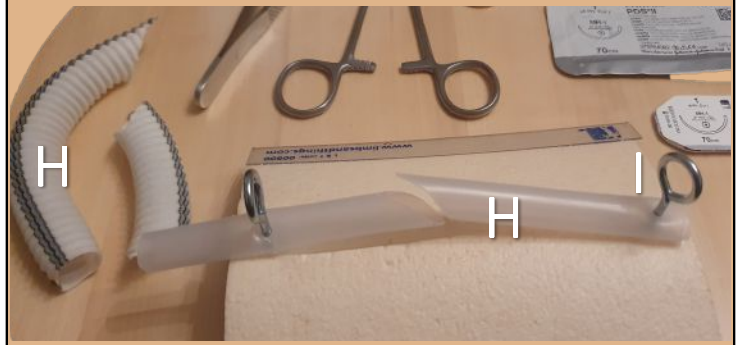


**Equipment:**

A. Sutures (preferably Prolene 3.0)
B. Toothed Forceps
C. Needle Holder
D. Suture Scissors

H. Vascular Model (or plastic tubing 8mm internal diameter)
I. Pad + Pins (to pin down vascular model)

Links to purchase:
A, B, C, D and E) https://www.kitsofmedicine.com/product/suture-kit/ – £31.20
F) https://groceries.asda.com/product/jelly-chewy-sweets/haribo-balla-stixx-strawberry/1000039942689 – £0.90
G and I) https://www.amazon.co.uk/Bulk-Hardware-BH01111-Chrome-Shouldered/dp/B00JIQPSOK/ref=sr_1_10?keywords=screw+hooks&qid=1640648619&sr=8-10 – £1.90
H) https://www.amazon.co.uk/dp/B08ZJL9JHW/ref=twister_B08ZJLQ2YW?_encoding=UTF8&psc=1 – £2.77
