# Supplementary material for: Can surgical skills be taught using technological advances online? A comparative study of online and face-to-face surgical skills training
Source: Surg Endosc. 2022 Mar 7;36(6):4631–7. doi: 10.1007/s00464-022-09170-5 (PMC9085701; doi:10.1007/s00464-022-09170-5)
Supplement: Supplementary file 3 — Supplementary file3 (PDF 52 kb) [file 464_2022_9170_MOESM3_ESM.pdf]

FSS Feedback

Are you receiving this teaching online or face-to-face (F2F)

- ○ Online (1)
- ○ F2F (2)

Year of Study

- ○ 1st Year (1)
- ○ 2nd Year (2)
- ○ 3rd Year (3)
- ○ 4th Year (4)
- ○ 5th Year (or Final Year) (5)
- ○ Intercalating Year (6)
- ○ FY1 (7)
- ○ FY2 (8)
- ○ ACF (9)
- ○ Other (Medical) (10)
- ○ Other (non-medical) (11)

Please rate your perceived competence in:

	0 1 2 3 4 5 6 7 8 9 10
Suturing ()	
'Scrubbing in' for theatre safely ()	
Surgical knot tying ()	
Knowing how to score competitively in CT/ST3 application ()	
Repairing a torn tendon ()	

Identifying the difference between forceps, artery clamps and needle-holder ()	
Performing a vascular anastomosis ()	

How certain are you in:

0 1 2 3 4 5 6 7 8 9 10

Pursuing a career in surgery ()	

Which style of conference do you think is best suited to teach basic surgical skills?

- ○ Online (1)
- ○ In-person (2)

What did you most enjoy about FSS 2021

____

____

____

____

____

What did you least enjoy about FSS 2021

____

____

____

____

____

In which of the following were you most satisfied with

- ☐ Live Workshops (suturing, tendon repair and anastomosis)
- ☐ Pre-recorded Workshop

☐ Lectures

Please rate:

How likely are you to recommend an online FSS course to a friend or colleague?
How satisfied were you with the Hopin platform?

Unlikely/Unsatisfied Very Likely/Satisfied

0 1 2 3 4 5 6 7 8 9 10

Q10 Did you attend a workshop breakout room?

◯ Yes

◯ No

Please rate your agreement with the following statements:

	No<br>0	Somewhat<br>1	Yes<br>2
Was the teaching level of the course clearly defined? ()			
Were the delivery methods appropriate for achieving the learning outcomes? ()			
Were the learning outcomes of this course clearly defined? ()			
Was this course held in an environment which was suitable for achieving the course learning outcomes? ()			
Was the structure of the course appropriate for achieving the learning outcomes? ()			
Was the number of demonstrators sufficient to support achievement of the learning outcomes? ()			
Did the demonstrators present the appropriate level of knowledge and skills to effectively deliver the learning outcomes? ()			

If you have any further comments, please include them below

A few quick questions about the breakout rooms

How satisfied were you with the breakout room in general?

○ Extremely satisfied

○ Somewhat satisfied

○ Neither satisfied nor dissatisfied

○ Somewhat dissatisfied

○ Extremely dissatisfied

How satisfied were you with the demonstrator?

○ Extremely satisfied

○ Somewhat satisfied

○ Neither satisfied nor dissatisfied

○ Somewhat dissatisfied

○ Extremely dissatisfied

Please rate:
Unlikely/Unsatisfied Very Likely/Satisfied 0 1 2 3 4 5

6 7 8 9 10

Did you find the demo's feedback useful ()	
Were the demo's instructions clear to understand and follow ()	
Were the demo's answers to your questions satisfactory ()	
Did the online teaching match the quality of an in-person session, in terms of educational value and efficacy ()	

A few quick questions about the stage demonstrations

How satisfied were you with the workshop demos on the stage?

○ Extremely satisfied

○ Somewhat satisfied

○ Neither satisfied nor dissatisfied

○ Somewhat dissatisfied

○ Extremely dissatisfied

How satisfied were you with the demonstrator?

○ Extremely satisfied

○ Somewhat satisfied

○ Neither satisfied nor dissatisfied

○ Somewhat dissatisfied

○ Extremely dissatisfied

Please rate:
Unlikely/Unsatisfied Very Likely/Satisfied 0 1 2 3 4 5

6 7 8 9 10

Did you find the demo's interaction with delegates useful ()	
Were the demo's instructions clear to understand and follow ()	
Were the demo's answers to the questions satisfactory ()	
Did the online teaching match the quality of an in-person session, in terms of educational value and efficacy ()	
