# Supplementary material for: Can surgical skills be taught using technological advances online? A comparative study of online and face-to-face surgical skills training
Source: Surg Endosc. 2022 Mar 7;36(6):4631–7. doi: 10.1007/s00464-022-09170-5 (PMC9085701; doi:10.1007/s00464-022-09170-5)
Supplement: Supplementary file 4 — Supplementary file4 (PDF 427 kb) [file 464_2022_9170_MOESM4_ESM.pdf]

Student Workshop Score

Please fill in this assessment for your students

*Required

Online or F2F

Choose

Suturing technique

	Yes	No
Needle held at one–third off length away from the base off the needle	○	○
Distance between sutures equals twice the thickness off wound	○	○
Does not touch needle with hand	○	○
Right angle entry to tissue on first side	○	○
Distance from wound equals	○	○
	○	○

thickness off tissue on first side

Distance from wound equals thickness off tissue on second side

Right angle exit from second side

Knott: 2 throws in one direction

Knott: 1 throw in the other direction

Knott: 1 throw in the initial direction

Knott: tied with adequate tension

Knott: lays to one side

Length off suture ends adequate

Whole suture lies at right angle to wound

Tendon Repair Technique

	Yes	Partially	No
Sutures places parallel to tendon ((1cm from edges))	◯	◯	◯
Hand tied knot effectively	◯	◯	◯
Uses needle holder, not fingers, to handle needle	◯	◯	◯
Suture bites not too close to edge off tendon	◯	◯	◯
Tendon edges abutting, not gaping or bunching	◯	◯	◯

Anastomosis Technique

	Yes,	Partially	No
Correct arteriotomy	○	○	○
Matches cut end to arteriotomy size	○	○	○
Continuous repair performed	○	○	○
Choose appropriate sutures	○	○	○
Uses needle holder not fingers to handle needle	○	○	○
Passes needle from inside–outside off artery	○	○	○
Suture bites equidistant from both edges off vessels	○	○	○
Sutures spaced evenly	○	○	○
Everted edges	○	○	○
Hand tied knot	○	○	○
Sutures knotted at midpoint off arteriotomy not apex	○	○	○
Anastomosis	○	○	○

completed in time

Submit
