# Supplementary material for: Can surgical skills be taught using technological advances online? A comparative study of online and face-to-face surgical skills training
Source: Surg Endosc. 2022 Mar 7;36(6):4631–7. doi: 10.1007/s00464-022-09170-5 (PMC9085701; doi:10.1007/s00464-022-09170-5)
Supplement: Supplementary file 5 — Supplementary file5 (PDF 266 kb) [file 464_2022_9170_MOESM5_ESM.pdf]

| Face-to-Face | |
|---|---|
| **Country** | **Attendees** |
| United Kingdom | 20 |
| **Total** | **20** |

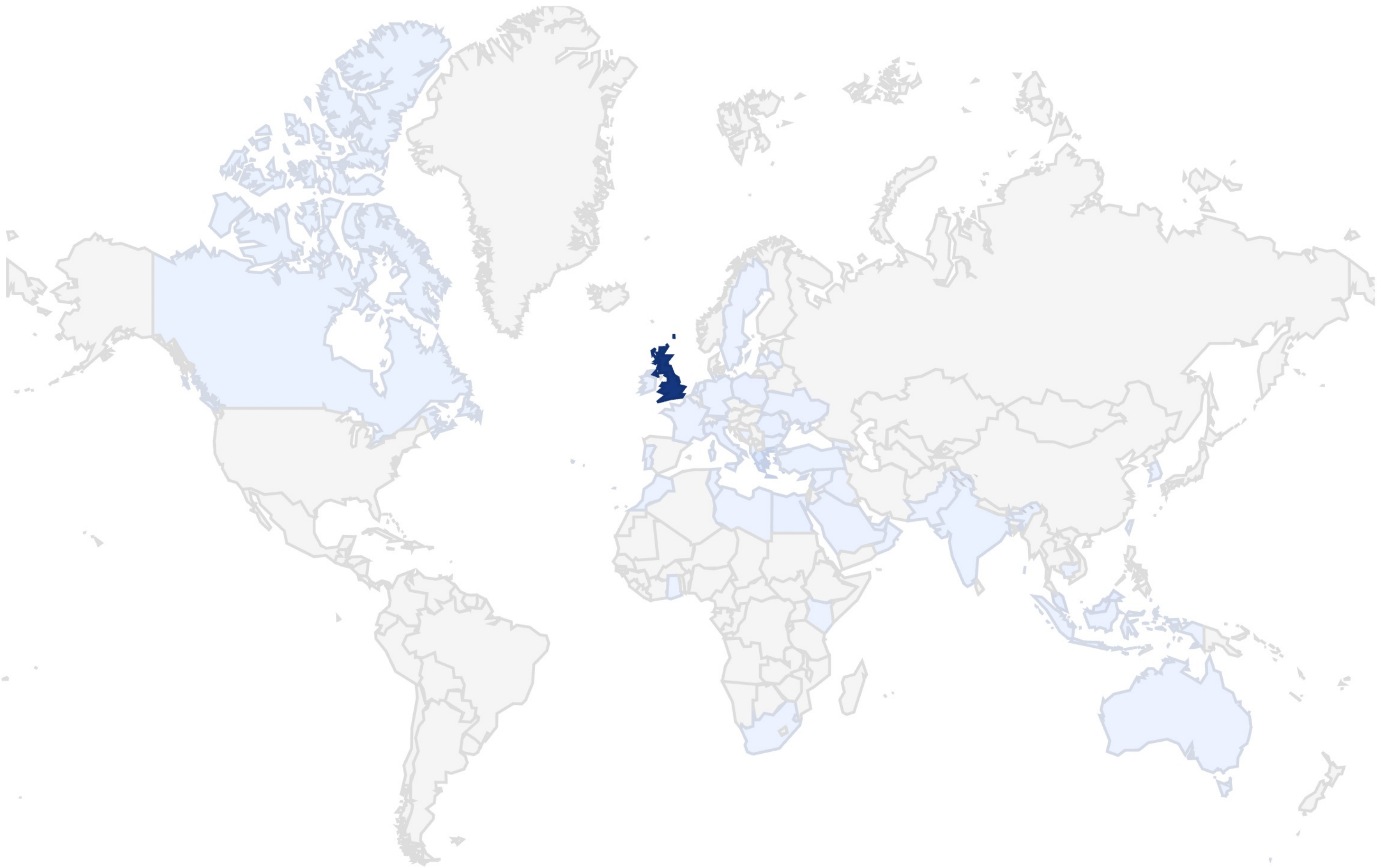

| Online | |
|---|---|
| **Country** | **Attendees** |
| Albania | 1 |
| Australia | 1 |
| Bahrain | 1 |
| Bosnia and Herzegovina | 1 |
| Bulgaria | 2 |
| Cambodia | 1 |
| Canada | 3 |
| Cyprus | 11 |
| Czechia | 2 |
| Egypt | 11 |
| France | 2 |
| Georgia | 1 |
| Greece | 39 |
| Hong Kong | 5 |
| Hungary | 1 |
| India | 6 |
| Indonesia | 1 |
| Iraq | 1 |
| Ireland | 5 |
| Italy | 9 |
| Kenya | 1 |
| Latvia | 1 |
| Lebanon | 1 |
| Libya | 1 |
| Malaysia | 13 |
| Malta | 9 |
| Oman | 1 |
| Pakistan | 1 |
| Poland | 11 |
| Portugal | 2 |
| Republic of Lithuania | 5 |
| Romania | 4 |
| Saudi Arabia | 4 |
| Singapore | 4 |
| South Africa | 1 |
| Spain | 1 |
| Taiwan | 1 |
| Tunisia | 1 |
| Turkey | 2 |
| Ukraine | 4 |
| United Arab Emirates | 4 |
| United Kingdom | 377 |
| **Total** | **553** |
